# The extent and serial pattern of interfractional variation in patients with whole pelvic irradiation: a study using a kilovoltage orthogonal on‐board imager

**DOI:** 10.1120/jacmp.v13i2.3636

**Published:** 2012-03-08

**Authors:** Won Sup Yoon, Dae Sik Yang, Jung Ae Lee, Suk Lee, Young Je Park, Chul Yong Kim

**Affiliations:** ^1^ Department of Radiation Oncology Korea University College of Medicine Seoul Republic of Korea

**Keywords:** interfractional variation, on‐board imagers, pelvic irradiation

## Abstract

The purpose of this study is to assess the extent and serial pattern of setup error of conventional fractionated whole pelvic irradiation using a kilovoltage on‐board imager. The daily on‐board images of 69 patients were matched with the digitally reconstructed radiographs of simulation on the basis of pelvic bony structure. The shifts along x‐ (lateral), y‐ (longitudinal), and z‐ (vertical) axes, and the 3D vector, were measured. The shift between an origin of the first fraction and each fraction $\left(\Delta {\text{shift}}^{1\text{st}}\right)$ and the shift between an isocenter of simulation and each fraction $\left(\Delta {\text{shift}}^{\text{Sim}}\right)$ were calculated. To evaluate serial changes, the shifts of each fraction were classified into four consecutive sessions, and an ANOVA and chi‐square test were used. The systematic error of the $\Delta {\text{shift}}^{\text{Sim}}$ and $\Delta {\text{shift}}^{1\text{st}}$ were 2.72 and 1.43 mm along the x‐axis, 2.98 and 1.28 mm along the y‐axis, and 4.26 and 2.39 mm along the z‐axis, respectively. The $\Delta {\text{shift}}^{\text{Sim}}$ and $\Delta {\text{shift}}^{1\text{st}}\geq 5\;\text{mm}$ of the 3D vector occurred in 54.3% and 23.1%, respectively. The recommended margins to cover setup error in case of using $\Delta {\text{shift}}^{1\text{st}}$ were 3.81, 3.54, and 6.01 mm along x‐, y‐, and z‐axes, whereas those using $\Delta {\text{shift}}^{\text{Sim}}$ were 6.39, 6.95, and 9.95 mm, respectively. With the passage of time, the $\Delta {\text{shift}}^{1\text{st}}\geq 5\;\text{mm}$ of 3D vector and along any axis in supine setup increased from 14.1% for first session to 22.5% for fourth session (p=0.027) and from 10.8% to 18.5% (p=0.034), respectively. In prone setup, first session was better than others in the $\Delta {\text{shift}}^{1\text{st}}\geq 5\text{mm}$ of 3D vector and along any axis. It is expected that the correction using the on‐board images on the first fraction improves geometrical uncertainties and reduces the margin for target coverage. Daily continuous OBI follow‐up during conventional fractionated pelvic irradiation can increase the reproducibility and be more effective in the late period.

PACS number: 87.55.km

## I. INTRODUCTION

Geometrical uncertainties of radiation therapy are caused by patient setup variation, organ motion and deformation, and machine‐related error.^(^ ^1^ ^,^ ^2^ ^)^ An electronic portal imaging device (EPID) is capable of measuring patient setup error.^(^ ^3^ ^–^ ^6^ ^)^ Recently, an online imaging device allows for accurate beam delivery after adjustment for fraction‐to‐fraction error. Kilovoltage (kV) or megavoltage (MV) fan‐ or cone‐beam computed tomography (CT) and kV orthogonal images are available to measure and correct interfractional variations. The advantage of online correction using CT is to match the images according to not only bony structure, but also soft tissue. Therefore, adaptive radiation therapy considering organ motion and deformity is possible.^(^ ^7^ ^–^ ^9^ ^)^ However, it would add heavy loading to the machine, require additional manpower, and be time‐consuming. In addition, the clinical significance to reduce margin recurrence and complications is unknown.

A kV orthogonal image from an on‐board imager (OBI) provides diagnostic quality images and can be compared with digital reconstruction radiographs (DRRs) originating in virtual CT simulation according to bony structure with 2D/2D match. kV images are able to improve interobserver and intraobserver variability compared to MV images.^(10)^ The volume of whole pelvic irradiation encompasses primary tumor or the tumor bed and regional lymph nodes. A four‐field (anterorposterior (AP), posterioanterior (PA) and both laterals) or three‐field (PA and both laterals) technique is considered to be the conventional method. Thus, delivery form the orthogonal directions is conducted. The advantages of kV orthogonal images for whole pelvic irradiation are that the direction for the acquisition of images is identical to the real beam delivery direction, and the pelvis has an enough bony structure including the sacrum and pelvic bone (consisting of the iliac, ischial, and pubic bones). Therefore, accurate 2D/2D match using an OBI is readily conducted.

Our institution has one‐year experience in applying 2D/2D match using a kV orthogonal OBI for patients undergoing whole pelvic irradiation. The main aim of this study was to assess the extent and pattern of setup error of whole pelvic irradiation. In addition, our serial data collected during the whole irradiation period, which requires 4–6 weeks, can evaluate the pattern of interfractional variations in relation to the passage of time.

## II. MATERIALS AND METHODS

### A. Patients

Our study was a prospective study conducted between April 2009 and March 2010, and included patients who received whole pelvic irradiation at our institution. Sixty‐nine of 70 patients were evaluated. One patient was excluded from the analysis because of the discontinuation of treatment over two weeks after the 9th fraction. The other long‐term discontinuations of two patients occurred after the 22nd and 23rd fractions. The fractions after discontinuation were excluded from analysis. The median elapsed period of radiation therapy was 35 days (range: 20–48 days). The median age was 57 years (range: 37–89 years). One patient with recurrent rectal cancer in the pelvic cavity had an ECOG score of 2. The other ECOG scores were 0 or 1. The most common primary disease was rectal cancer (N=30), followed by cancer of uterine cervix (N=22), and uterine corpus (N=10). The patients with rectal cancer and sarcoma were placed in the prone position. One patient complained of discomfort in the prone position, therefore the position was changed to supine after the 9th fraction. The patient characteristics are summarized in Table 1.

**Table 1:** Patient characteristics.

*Characteristic*	*Number*
Sex	
male: female	30:39
Age (years)	
median (range)	57 (37–89)
Primary disease	
uterine cervix	22
uterine corpus	10
rectum	30
prostate	5
bladder	1
sarcoma	1
Position	
supine	38
prone	30
prone ρ supine	1
ECOG performance	
0–1	68
2	1
Treatment aim	
adjuvant	32
definite	37
Concurrent chemotherapy	
yes: no	50:19
Number of fraction	
28:25:24:23:22:20:16:15	22:30:4:2:8:1:1:1
Elapsed time (days)	
median (range)	35 (20–48)

### B. Verification technique

**Figure 1:**
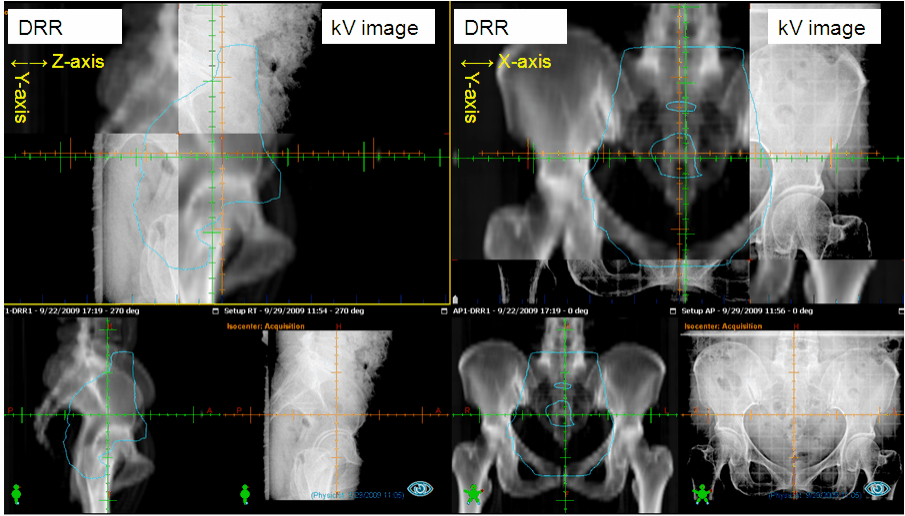
Example of kilovoltage on‐board imaging. These orthogonal images of the gantry at 0° (right) and 270° (left) were taken before the beam delivery and matched to the digitally reconstructed radiograph (DRR) according to bony structure. The green and orange lines are alignment of simulation and each fraction, respectively. In this example, 4, 5, and 7 mm shifts from the alignment of simulation were developed in the x‐, y‐, and z‐axes, respectively.

Virtual simulation using a CT simulator (GE Healthcare, Waukesha, WI) was performed with a slice thickness of 5 mm and the origin of CT simulation was marked in the body of the patient. The setup position was selected by physician preference. None of the immobilization device was used for supine setup. Prone setup involved a commercially available vacuum bag as a belly board. CT images were transferred to an Eclipse treatment planning system (Varian Medical System, Palo Alto, CA), and gross and clinical target volumes (CTVs) and organs at risk were drawn. The optimized isocenter of simulation was determined by the individual characteristic. The isocenter of simulation was inconsistent with the origin of CT simulation in some of the patients. A four‐ or three‐field technique was applied to planning. On the first fraction, a setup was performed according to the isocenter of simulation. The kV orthogonal images (gantry, 0° and 270°/90°), using an OBI that is mounted on our Trilogy linac (Varian Medical System) and employs a G242 X‐ray tube and a PaxScan 4030CB amorphous silicon imaging panel, were acquired and matched with the DRRs of CT simulation. Our linac and Varian Exact Couch (Varian Medical System) were installed in our institution in June 2008. A Customer Acceptance Procedure was provided by vendor and passed. A periodic quality assurances including daily, monthly, and annually mechanical and imaging checks corresponding with the reports of AAPM Task Group 104 and 142 have been performed, and the tolerance values for intensity‐modulated radiation therapy were satisfied.^(^ ^11^ ^,^ ^12^ ^)^ The accuracy of the isocenter of our OBI was less than 1.5 mm. To reduce interobserver variation, two experienced therapeutic radiologists and one radiation oncologist determined an optimized shift on the basis of pelvic bony structure (i.e., the pubis symphysis, obturator foramen, ilium, sacroiliac joint, and sacrum (Fig. 1). If shifts occurred, re‐image acquisition and confirmation of consistency were performed. The origin of the first fraction was re‐marked in the body of the patient. Localizing laser in linac room was used to assist this procedure. The setup after the first fraction was based on the origin of the first fraction; the daily kV orthogonal images were acquired and matched with simulation DRR, and then correction (if applicable) was taken.

### C. Data analysis

The optimized shifts along x‐, y‐, and z‐ axes were measured (Fig 1) through an off‐line review. The 3D vector of each shift was calculated using the following equation: $3D\;\text{vector}=\surd [\Delta x^{2}+\Delta x^{2}+\Delta z^{2}]$.^(13)^

The shift between an origin of the first fraction and each fraction $\left(\Delta {\text{shift}}^{1\text{st}}\right)$ and the shift between an isocenter of simulation and each fraction $\left(\Delta {\text{shift}}^{\text{Sim}}\right)$ were used. The $\Delta {\text{shift}}^{1\text{st}}$ was surveyed and the $\Delta {\text{shift}}^{\text{Sim}}$ was calculated considering the variation between a simulation and the first treatment. The $\Delta {\text{shift}}^{1\text{st}}$ was used for the analyses of setup position and the passage of time.

Systematic errors were defined as the mean deviation of the geometry between the fractionated treatment and an isocenter of the first fraction or simulation. Random errors were defined as the mean deviation that occurred by fraction‐to‐fraction variations.^(14)^ The estimation of various errors was based on measurements in a population of patients. The overall mean error, M, the standard deviation (SD) of the systematic error, ∑, and the SD of the random error, σ, were determined. The M is the mean of all means. The ∑ is the SD of M. The σ is the root mean square of the individual SD.^(15)^

To evaluate serial changes in interfractional variations, the shifts of each fraction were classified into four sessions. One session consisted of five consecutive fractions. The first, second, and third fractions were excluded from the analysis in association with the passage of time because of the probability of the bias according to excessive instability for the setup and the affect on concurrent chemotherapy. Therefore, each session was defined as follows:

- first session, from the 4th fraction to the 8th fraction
- second session, from the 9th fraction to the 13th fraction
- third session, from the 14th fraction to the 18th fraction
- fourth session, from the 19th fraction to the 23rd fraction

Patients with information in at least four fractions in each session were analyzed in a study related with the systematic errors (∑) and the random errors (a).

### D. Statistical analysis

Statistical analysis was performed on the interfractional variations using SPSS 12.0 (SPSS Inc., Chicago, IL). For each of the shifts along the x‐, y‐, and z‐axes and 3D vector, descriptive analysis was conducted. The values at 90%, 95%, and 99% from zero (no shift) and the occurrence rate of errors ≥5mm were calculated using absolute values. In addition, the recommended margins from the CTV to cover the setup errors were calculated using the following equation: margin=2∑+0.7σ. For normally distributed deviations and in absence of significant rotational deviations, this equation satisfied the assumption that, on average, a high percentage of the CTV volume (99%) should receive a high dose (95%) as demonstrated in lung, prostate, and uterine cervix cancer.^(16)^ To compare two different mean values, variations and occurrence rates of error, a t‐test, F‐test, and chi‐square test were used, respectively. To evaluate the significance of serial change in interfractional variations, a one‐way ANOVA test was used. A chi‐square test was used for the change of the occurrence rate of error. A p<0.05 was considered statistically significant.

## III. RESULTS

### A. Extent and pattern of interfractional variations

One thousand seven hundred and thirty‐four (1734) fractions were analyzed. The maximum error was 21 mm. The ∑s of the $\Delta {\text{shift}}^{\text{Sim}}$ and $\Delta {\text{shift}}^{1\text{st}}$ were 2.72 and 1.43 mm along the x‐axis, 2.98 and 1.28 mm along the y‐axis, and 4.26 and 2.39 mm along the z‐axis, respectively. The Ms of 3D vector were 5.78 and 3.30 mm for the $\Delta {\text{shift}}^{\text{Sim}}$ and $\Delta {\text{shift}}^{1\text{st}}$, respectively. The σ of the $\Delta {\text{shift}}^{\text{Sim}}$ and $\Delta {\text{shift}}^{1\text{st}}$ were 1.36 and 1.35 mm along the x‐axis, 1.41 and 1.40 mm along the y‐axis, and 2.04 and 2.02 mm along the z‐axis, respectively (Table 2).

**Table 2:** The interfractional variation of whole pelvic irradiation in 69 patients.

	*x‐axis*	*y‐axis*	*z‐axis*	*3D Vector*
Between an origin of simulation and each fraction $\left(\Delta {\text{shift}}^{\text{Sim}}\right)$				
range (mm)	−20−10	−15−14	−21−15	0 ‐ 21.2
M (mm)	−0.16	−1.06	−1.67	5.78
∑ (mm)	2.72	2.98	4.26	
σ (mm)	1.36	1.41	2.04	
value at 90%: 95%: 99% from no shift (zero) (mm)	4: 6: 15	6: 7: 10	8: 10: 15	10.1: 12: 18
occurrence rate of error ≥5mm (%)	8.1	18.1	34.4	54.3 (50.1)^a^
Between an origin of the first fraction and each fraction $\left(\Delta {\text{shift}}^{1\text{st}}\right)$				
range (mm)	−9−18	−8−10	−12−15	0 ‐ 18.3
M (mm)	0.03	−0.19	−0.73	3.30
∑ (mm)	1.43	1.28	2.39	
σ (mm)	1.35	1.40	2.02	
value at 90%: 95%: 99% from no shift (zero) (mm)	3: 4: 7	3: 4: 6	5: 6: 11	6.4: 8.1: 12.1
occurrence rate of error ≥5mm (%)	4.6	4.3	14.3	23.1 (20.5)^a^

a The occurrence rate of error ≥5mm along any axis in each fraction.

x‐axis= lateral direction; y‐axis= longitudinal direction; z‐axis= vertical direction; $3D\;\text{vector}=\surd (\Delta x^{2}+\Delta y^{2}+\Delta z^{2})$; ∑= population standard deviation of the systematic shifts; σ= the root mean square of the random errors.

The $\Delta {\text{shift}}^{\text{Sim}}$ and $\Delta {\text{shift}}^{1\text{st}}$ ≥5mm along the x‐, y‐ and z‐axes occurred in 8.1% and 4.6%, 18.1% and 4.3%, and 34.4% and 14.3%, respectively. The $\Delta {\text{shift}}^{\text{Sim}}$ and $\Delta {\text{shift}}^{1\text{st}}$ ≥5mm of the 3D vector occurred in 54.3% and 23.1%, respectively (Table 2). The occurrence rate of error ≥5mm along any axis in each fraction was 50.1% and 20.5% for the $\Delta {\text{shift}}^{\text{Sim}}$ and $\Delta {\text{shift}}^{1\text{st}}$, respectively.

The $\Delta {\text{shift}}^{1\text{st}}$ values at 95% from zero were 4, 4, 6, and 8.06 mm for the x‐, y‐ and z‐axes and 3D vector, and the values at 99% were 7, 6, 11, and 12.08 mm, respectively. For the $\Delta {\text{shift}}^{\text{Sim}}$ values at 95% and 99% from zero, 6 and 15 mm along the x‐axis, 7 and 10 mm along the y‐axis, 10 and 15 mm along the z‐axis, and 12 and 18 mm along the 3D vector were observed, respectively (Table 2). The recommended margins to cover the setup error when using the OBI on the first day were 3.81, 3.54, and 6.01 mm along x‐, y‐, and z‐axes, respectively. These values nearly covered the $\Delta {\text{shift}}^{1\text{st}}$ values at 95% from zero. If the margins were calculated without consideration of the correction using the OBI, then 6.39, 6.95, and 9.95 mm along x‐, y‐, and z‐axes, respectively, are to be used.

The $\Delta {\text{shift}}^{\text{Sim}}$ was worse than the $\Delta {\text{shift}}^{1\text{st}}$ for the ∑ of the x‐, y‐, and z‐axes and the Ms of 3D vector (p<0.000). For the occurrence rate of error ≥5mm, similar tendency was also observed for the x‐, y‐, and z‐axes and 3D vector (p<0.000).

### B. Setup position

One patient who changed a position from prone to supine was excluded from the analysis. Sixty‐eight patients were evaluated. Our population can be differentiated according to setup position and immobilization device. One is supine setup without immobilization device (Supine), and other is prone setup with belly board (Prone). On the basis of $\Delta {\text{shift}}^{1\text{st}}$, the Ms of 3D vector were 2.97±1.32 and 3.71±2.16mm for Supine and Prone, respectively. The difference between the groups was insignificant (p=0.086). The extent of the ∑ and σ along x‐ and y‐axes were similar. However, Prone was worse than Supine in both the ∑ (p<0.000) and σ (p=0.001) for z‐axis (Table 3).

**Table 3:** The interfractional variation according to the patient position.

	*x‐axis*	*y‐axis*	*z‐axis*	*3D Vector*
Supine (N=37)				
M (mm)	−0.13	0.10	−0.82	2.97±1.32
∑ (mm)	1.44	1.21	1.52	
σ (mm)	1.52	1.39	1.72	
Prone (N=31)				
M (mm)	0.22	−0.53	−0.59	3.71±2.16
∑ (mm)	1.44	1.31	3.17	
σ (mm)	1.10	1.40	2.35	

x‐axis=lateraldirection; y‐axis=longitudinaldirection; z‐axis=verticaldirection; $3D\;\text{vector}=\surd (\Delta x^{2}+\Delta y^{2}+\Delta z^{2})$; ∑= population standard deviation of the systematic shifts; σ= the root mean square of the random errors.

### C. Serial pattern of interfractional variations

In Supine, the Ms of 3D vector were 2.78, 3.09, 2.92, and 2.98 mm for first, second, third, and fourth session, respectively. The ranges of the ∑s along x‐, y‐ and z‐axes were from 1.46 to 1.86 mm, from 1.35 to 1.82 mm, and from 1.82 to 1.99 mm, and the σ were from 1.24 to 1.48 mm, from 1.08 to 1.51 mm, and from 1.45 to 1.72 mm, respectively. With the passage of time, no significant pattern was observed (Table 4).

**Table 4:** The relation with the extent of interfractional variation and the passage of time according to the setup position.

	*1st session*	*2nd session*	*3rd session*	*4th session*	*p value*
Supine	N=37	N=37	N=37	N=36	
x‐axis					
M±∑(mm)	−0.27±1.48	−0.01±1.67.	−0.17±1.86	−0.01±1.46	0.879
σ (mm)	1.41	1.48	1.24	1.41	
y‐axis					
M±∑(mm)	−0.20±1.82	0.17±1.35	0.07±1.40	−0.20±1.54	0.981
σ (mm)	1.35	1.32	1.08	1.51	
z‐axis					
M±∑(mm)	−0.97±1.82	−0.78±1.91	−0.77±1.83	−0.66±1.99	0.864
σ (mm)	1.45	1.63	1.53	1.72	
3D Vector					
M (mm)	2.78	3.09	2.92	2.98	0.798
median (mm)	2.51	3.10	2.59	2.66	
Prone	N=31	N=31	N=30	N=28	
x‐axis					
M±∑(mm)	0.09±1.13	0.18±1.57	0.47±1.82	0.34±2.01	0.631
σ (mm)	0.93	0.99	0.89	0.80	
y‐axis					
M±∑(mm)	−0.49±1.13	−0.57±1.40	−0.74±1.56	−0.35±2.08	0.296
σ (mm)	1.31	1.25	1.20	1.38	
z‐axis					
M±∑(mm)	−0.80±3.51	−0.61±3.50	−0.62±3.66	−0.50±3.97	0.799
σ (mm)	2.26	2.24	2.07	1.95	
3D Vector					
M (mm)	3.51	3.81	4.01	4.22	0.739
median (mm)	2.85	3.21	3.27	3.48	

x‐axis=lateraldirection; y‐axis=longitudinaldirection; z‐axis=verticaldirection; $3D\;\text{vector}=\surd (\Delta x^{2}+\Delta y^{2}+\Delta z^{2})$; M= population mean of the systematic shifts; ∑= population standard deviation of the systematic shifts; σ= the root mean square of the random errors.

In Prone, the Ms of 3D vector were 3.51, 3.81, 4.01, and 4.22 mm for first, second, third, and fourth session, respectively. The ranges of the ∑s along x‐, y‐ and z‐axes were from 1.13 to 2.01 mm, from 1.13 to 2.08 mm, and from 3.50 to 3.97 mm, respectively. Although the serial deterioration was observed in the 3D vectors and the ∑s, the differences of extent were too minimal to have a statistical significance. The ranges of the σ were from 0.80 to 0.99 mm, from 1.20 to 1.38 mm, and from 1.95 to 2.26 mm, respectively and have no significant pattern with the passage of time (Table 4).

**Figure 2:**
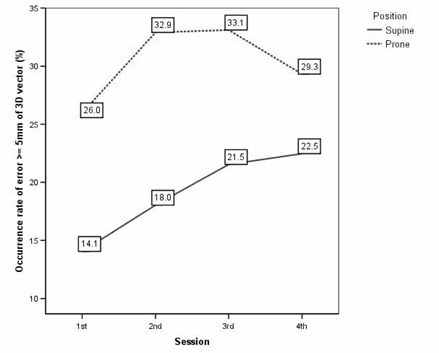
In patients with supine setup, interfractional variations between an origin of the first fraction and each fraction in terms of the occurrence rate of error ≥5mm of three dimensional displacements were worse with the passage of time (p=0.027). In patients with prone setup, 1st session was better than others. However, serial aggravation was not observed.

**Figure 3:**
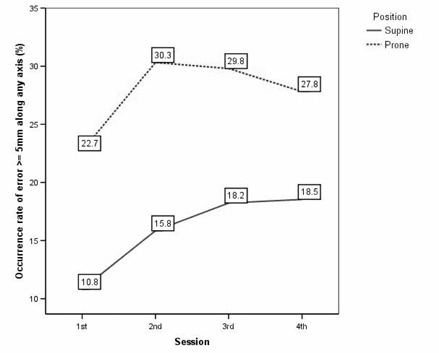
In patients with supine setup, interfractional variations between an origin of the first fraction and each fraction in terms of the occurrence rate of error ≥5mm along any axis were worse with the passage of time (p=0.034). In patients with prone setup, 1st session was better than others. However, serial aggravation was not observed.

In Supine, the $\Delta {\text{shift}}^{1\text{st}}$ ≥5mm of 3D vector increased from 14.1% for first session to 22.5% for fourth session (p=0.027; Fig. 2). Also, the occurrence rate of error ≥5mm along any axis increased from 10.8% for first session to 18.5% to fourth session (p=0.034; Fig. 3).

In Prone, the occurrence rate of error ≥5mm of 3D vector and along any axis for first session were 26.0% and 22.7%, respectively. These values were better than other sessions. However, the differences between other sessions were minimal; therefore, there was no significance of the occurrence rate of error ≥5mm of 3D vector (p=0.508, Fig. 2) and along any axis (p=0.348, Fig. 3) in relation with the passage of time.

## IV. DISCUSSION

Our study had a sizeable population of 69 patients and a total of 1734 fractions. All patients who had whole pelvic irradiation with a conventional fractionated schedule in our institution during one year were included in our study as a prospective method. The serial information on interfractional variations through kV orthogonal images enabled our study to analyze the relationship with interfractional variation and the passage of time.

Our study showed that the range of ∑ and σ in case of using the OBI on the first day was from 1.28 to 2.39 mm and from 1.35 to 2.02 mm, respectively. The setup error of the vertical axis was the most extensive. There have been a few studies for interfractional variation of the pelvis. Generally, the extent of setup error was less than 5 mm on the basis of bony structure, and the most deteriorated axis was the longitudinal or vertical direction.^(^ ^4^ ^–^ ^6^ ^,^ ^9^ ^,^ ^17^ ^–^ ^19^ ^)^ A study using the ExacTrac system (Brainlab AG, Feldkirchen, Germany) showed that the main breathing direction in pelvis was from dorsocaudal to anteriorcranial.^(20)^

Our study results confirmed that an error ≥5mm along any axis between an origin of the first fraction and each fraction occurred in approximately one fraction out of five fractions. If the OBI is applied to daily treatment, we expect that the occurrences of error are adequately controlled. However, a heavy loading by daily verification and clinical significance to suppress the margin recurrence and complication are undetermined.

We have recommended appropriate margins for patient setup variation. These recommendations were based on the confidence that the setup error between virtual simulation and the first treatment is successfully corrected by the kV orthogonal OBI. The appropriate margins along the x‐, y‐ and z‐axes were 3.81, 3.54 and 6.01 mm, respectively. In early phase study using the EPID, Mock et al.^(4)^ reported that planning target volume margins of 1 cm appeared to be sufficient, accounting for more that 95% of all deviations. Kaatee et al.^(18)^ considered both bony structure and cervical movement using the EPID and cervical marker, and recommended that the appropriate margins along the x‐, y‐, and z‐axes were 10, 12, and 12 mm, respectively. Ost et al.^(9)^ recommended a 6–8mm planning target volume margin using cone‐beam CT in prostate cancer. Santanam et al.^(21)^ presented a net radial margin of about 7 mm in gynecologic malignancies using planar kV and portal MV imaging and tomotherapy.

The setup variation with an isocenter of simulation as an origin was more extensive than the first‐day fraction, probably because of the mechanical error between the CT and linac and physical and psychological instability due to the patients' inexperience. Wu et al^.(22)^ reported that the first‐day correction using the kV cone‐beam CT reduced the systematic errors in prostate cancer. A study using the EPID showed that the early correction of the systematic errors increased the setup accuracy in brain, head and neck, and pelvis.^(3)^ A study using megavoltage CT reported that high‐frequency image guidance provided a reduction in setup errors.^(13)^ In our study, if a process with the kV orthogonal OBI omits or inaccurately proceeds, additional margins (2.58, 3.41, and 3.94 mm along x‐, y‐, and z‐axes, respectively) are required. We anticipate that the kV orthogonal OBI performs an effective role in verifying and correcting the patient setup error.

Patient position has been suggested as the factor related to the setup errors in pelvic irradiation.^(^ ^6^ ^,^ ^13^ ^)^ The setup position affects the immobilization tool and setup techniques. Also, our study selected the setup position according to physician preference, and thus presented uneven distribution between primary disease and the setup position. Therefore, our study performed subgroup analysis for position. Supine position was better than Prone position in the extent of the errors along z‐axis. Therefore, we separated patients into two groups in our study on the passage of time.

One of our chief objectives was the relationship with the interfractional variation and the passage of time. Because we were concerned about unexpected setup deviation in the early phase of treatment, the date from the first to third fractions was excluded from analysis for the passage of time. Supine position patients had increased occurrence rates of error ≥5mm with the passage of the time. Also, Prone position patients showed that the extent of the ∑s minimally increased with the passage of time, and the occurrence rate of errors ≥5mm in second, third, and fourth session was worse than first session. Our study showed that the probability of the deterioration could increase with the passage of time. Therefore, close observation using the frequent daily image verification will be needed in the late period of conventional scheduled pelvic irradiation.

Our study was based on the 2D/2D match using the kV orthogonal OBI. Accordingly, there were limits to comparing images with respect to soft tissue and analyze rotational factors. The information involving organ motion and deformity was unavailable. Although the AP/PA and lateral boundaries of radiation field might be more attributed to a distribution of lymph nodes than organs in conventional whole pelvic irradiation encompassing regional lymph nodes,^(23)^ it was a weak point in our study. Rotational errors in pelvic irradiation were presented in some articles. In studies based on MV fan‐beam images, the Ms±SDs of $0.315\pm 1.155^{\circ }$ (37 patients) and $0.5\pm 0.9^{\circ }$ (27 patients) were observed by Allen Li et al.^(19)^ and Zhou et al.,^(24)^ respectively. The latter presented the maximum error, ∑, and σ of 3.7°, 0.7°, and 0.7°, respectively. In a study based on film and EPID, ∑ and σ were 1.3° and 2.4° in AP direction and 2.1° and 4.4° in lateral direction, respectively.^(17)^ These extents of rotational error might be developed and disregarded in our study.

## V. CONCLUSIONS

In conclusion, the extent and probability of setup error between simulation and each treatment were more severe than between the first fraction and each individual fraction. In other words, geometrical uncertainties (i.e., patient setup variation and machine related errors can be improved when the OBI is applied for the isocenter correction on the first day). Therefore, it is expected that the OBI has a role to reduce the margin for target coverage. In addition, daily continuous OBI follow–up can correct the setup error ≥5mm in approximately 20%, thereby increasing the reproducibility. Our study observed increased occurrence of setup error related to the passage of time in one of our cohorts. Therefore, frequent and intensive verification will be more effective in the late period of conventional fractionated whole pelvic irradiation.
